# Melanin-embedded materials effectively remove hexavalent chromium (Cr^VI^) from aqueous solution

**DOI:** 10.1186/s12199-018-0699-y

**Published:** 2018-02-23

**Authors:** An Manh Cuong, Nguyen Thi Le Na, Pham Nhat Thang, Trinh Ngoc Diep, Ly Bich Thuy, Nguyen Lai Thanh, Nguyen Dinh Thang

**Affiliations:** 1 Department of Biochemistry and Molecular Biology, Faculty of Biology, VNU University of Science, Vietnam National University, 334 Nguyen Trai St., Thanh Xuan Dist, Hanoi, Vietnam; 2 High school for Gifted Students, VNU University of Science, Hanoi, Vietnam; 3 Institute for Environmental Science and Technology, Hanoi University of Science and Technology, Hanoi, Vietnam; 4 Key Laboratory of Enzyme and Protein Technology, VNU University of Science, Hanoi, Vietnam

**Keywords:** Cr^VI^, CMB, IMB

## Abstract

**Background:**

Currently, it is recognized that water polluted with toxic heavy metal ions may cause serious effects on human health. Therefore, the development of new materials for effective removal of heavy metal ions from water is still a widely important area. Melanin is being considered as a potential material for removal of heavy metal from water.

**Methods:**

In this study, we synthesized two melanin-embedded beads from two different melanin powder sources and named IMB (Isolated Melanin Bead originated from squid ink sac) and CMB (Commercial Melanin Bead originated from sesame seeds). These beads were of globular shape and 2–3 mm in diameter. We investigated and compared the sorption abilities of these two bead materials toward hexavalent-chromium (Cr^VI^) in water. The isotherm sorption curves were established using Langmuir and Freundlich models in the optimized conditions of pH, sorption time, solid/liquid ratio, and initial concentration of Cr^VI^. The FITR analysis was also carried out to show the differences in surface properties of these two beads.

**Results:**

The optimized conditions for isotherm sorption of Cr^VI^ on IMB/CMB were set at pH values of 2/2, sorption times of 90/300 min, and solid-liquid ratios of 10/20 mg/mL. The maximum sorption capacities calculated based on the Langmuir model were 19.60 and 6.24 for IMB and CMB, respectively. However, the adsorption kinetic of Cr^VI^ on the beads fitted the Freundlich model with *R*^2^ values of 0.992 for IMB and 0.989 for CMB. The deduced Freundlich constant, 1/n, in the range of 0.2–0.8 indicated that these beads are good adsorption materials. In addition, structure analysis data revealed great differences in physical and chemical properties between IMB and CMB. Interestingly, FTIR analysis results showed strong signals of –OH (3295.35 cm^− 1^) and –C=O (1608.63 cm^− 1^) groups harboring on the IMB but not CMB. Moreover, loading of Cr^VI^ on the IMB caused a shift of broad peaks from 3295.35 cm^− 1^ and 1608.63 cm^− 1^ to 3354.21 cm^− 1^ and 1597.06 cm^− 1^, respectively, due to –OH and –C=O stretching.

**Conclusions:**

Taken together, our study suggests that IMB has great potential as a bead material for the elimination of Cr^VI^ from aqueous solutions and may be highly useful for water treatment applications.

## Background

Currently, environmental pollution caused by rapid industrialization and technological advances is a worldwide problem. It is recognized that water polluted with toxic heavy metals can have serious effects on human health [1, 2]. There are many types of materials which have been being used to remove heavy metals from aqueous effluents; these include activated carbon, plant-leaf materials, chitosan gel, and hydrotalcite [3, 4]. However, these materials are not fully effective nor cost efficient.

Chromium is considered to be one of the key contaminants in the wastewaters of many industries, such as plating-electroplating, dying-pigmenting, film-photography, leathering and mining. Although both hexavalent chromium (Cr^VI^) and trivalent chromium (Cr^III^) are predominant species in industrial effluents, the Cr^VI^ is more toxic than Cr^III^. More seriously, the Cr^VI^ is considered as a mutagenic agent, which may cause adverse public health problems [1, 5].

Melanin is synthesized in humans, animals, invertebrate animals, bacteria, and fungi by oxidation of phenol or indole compounds [6–9]. Besides its role in pigmentation, melanin has many other important biological functions; it serves as an electron transporter, ion balancer, free radical acceptor as well as antioxidant, antibacterial, antivirus, and anticancer agent [6, 9]. Thus, melanin has been widely considered to be a potential material for use in various industries including agriculture, pharmacy, medicine, and cosmetics [6–9].

Recently, melanin powder (but not melanin bead) has also been examined for its ability to eliminate heavy metal ions (e.g., lead, cadmium, copper, and ferrous) in aqueous solutions [10–12]. Generally, for removing of heavy metal ions, a material in powder form should have very high sorption capacity. However, there is no guarantee that a high sorption capacity for the material exists in bead form [3, 4]. For practical conditions, such as in drinking water treatment, the bead form (rather than in powder form) of a material is the most popular and suitable form to avoid the possibility of being stuck when water flow passes through the material column. To date, there has been no study evaluating the use of melanin originated from squid ink sacs for removal of chromium ions, although a previous report showed that melanin secreted from *Aureobacidium pullulans* could also adsorb Cr^VI^ from waste water [13]. However, the different source of melanin may have big different capacity in removing of Cr^VI^ ion. In this study, we used two different melanin sources: one was isolated from squid ink sacs, which are considered as waste material of seafood processing companies and named as IMB (Isolated Melanin Bead), and the other was derived from sesame seeds (purchased from Xi’an Green Spring Technology Co., LTD, China) and named as CMB (Commercial Melanin Bead). These two melanin powders were used to make melanin-embedded beads for investigating their abilities to remove hexavalent chromium ions (Cr^VI^). This study also aimed to compare the capacity of Cr^VI^ uptake by the two melanin-embedded beads. Comparisons were made by examining differences in their physical and chemical properties due to their different source of origin.

## Methods

### Melanin isolation from squid ink sacs

The method used for isolating melanin has been described previously [14]. Briefly, squid ink sacs collected from the seafood company were broken down to collect ink liquid. This liquid (50 g) was dissolved into 200 mL of 0.5 M HCl. The mixture was then sonicated for 15 min in a sonicator followed by stirring for 30 min. The mixture was then incubated at 4 °C for 48 h before centrifuging at 10,000 rpm at 5 °C for 15 min to collect the pellet. The pellet was washed with acetone for three times then washed with distilled water for three times. The melanin pellet was dried at 60 °C, grinded, sieved through 150 μm, and then stored at room temperature.

### Method for making spherical melanin-embedded beads

Melanin beads were made according to a previously published protocol [15]. Briefly, melanin powder was embedded using sodium alginate as a cohesion agent. Sodium alginate was dissolved in 20 mL of distilled water and incubated in a water bath incubator at 70 °C to completely dissolve it before adding 5 g of melanin powder with continuous stirring. The mixture solution was drawn into a syringe and then eluted drop by drop into CaCl_2_ solution (5%) to create beads with spherical form and with a diameter of 2–3 mm. Next, the melanin beads were dried out and dipped into 5% CaCl_2_ solution for 24 h before washing with distilled water for three times and drying to unchanged weight.

### Fourier-transform infrared analysis

Infrared spectra of the material beads were obtained using a Fourier-transform infrared spectrometer (FTIR Affinity - 1S, SHIMADZU, Kyoto, Japan) [16].

### Microscope analysis

Morphology and purification of melanin powder isolated from squid ink sac was investigated by scanning electron microscope, model NANOSEM450 (Netherlands), and surface property of melanin bead was examined under Carlzeiss stereo-microscope, model stemi SV2000 (Germany).

### Sorption experiments and Cr^VI^ analytical methods

**Fig. 1:**
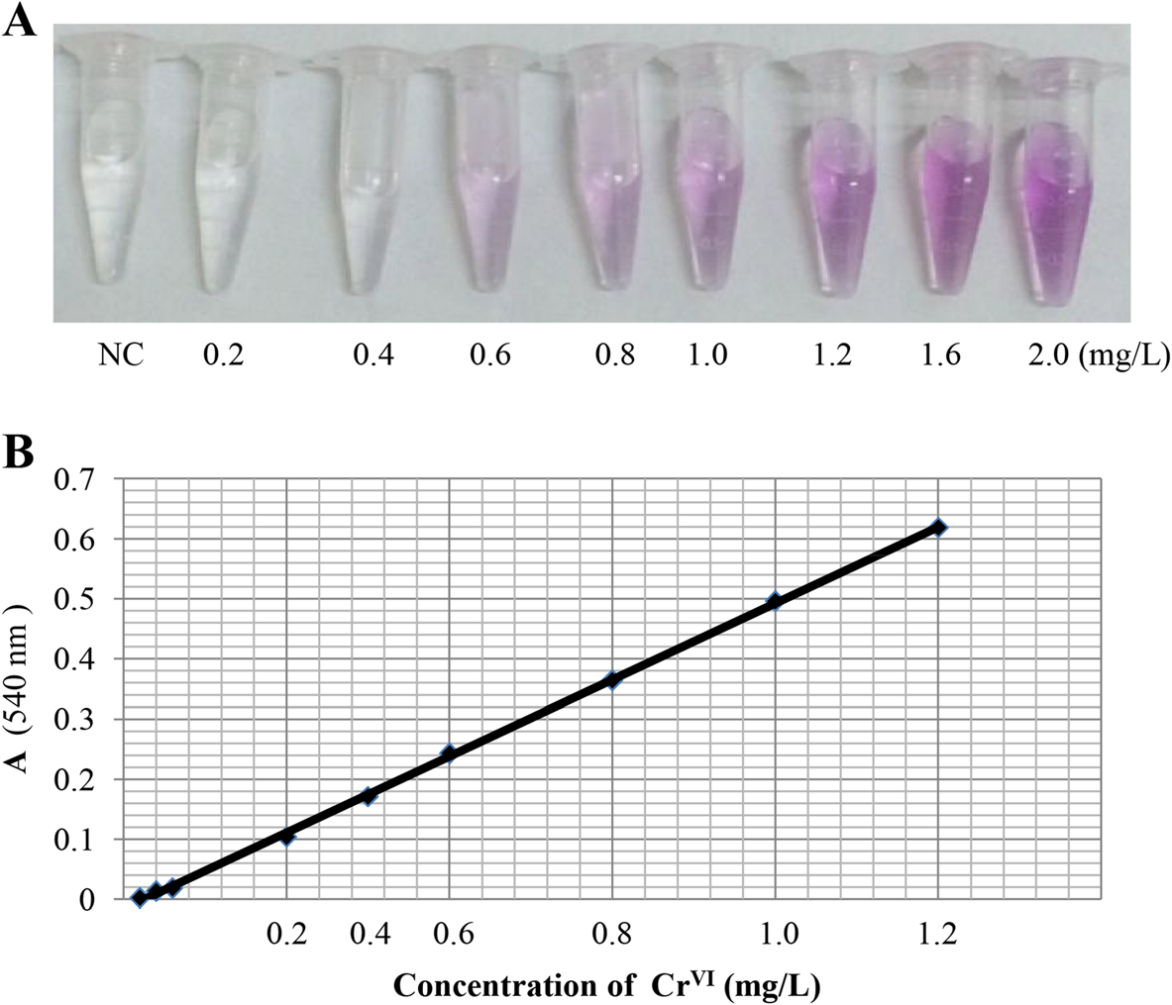
Purple-violet colored complex of (Cr^VI^-diphenyl cacbazide) at different concentrations (**a**) and standard curve for Cr^VI^ analysis measured by spectrophotometer (**b**) were presented

Experiments were conducted at room temperature. Batch equilibrium sorption experiments were carried out in 250 mL Erlenmeyer flasks containing potassium dichromate (K_2_Cr_2_O_7_) solutions (100 mL) of known concentrations (varying from 5 to 200 mg/L). Melanin was added into the K_2_Cr_2_O_7_ solution with various ratios of solid/liquid and placed on a shaker at 200 rpm for various time settings. The solution was then centrifuged at 10,000 rpm for 10 min. In the acidified medium, Cr^VI^ reacted with diphenyl carbazide to form a purple-violet colored complex. The concentration of Cr^VI^ in the supernatant was determined colorimetrically using a spectrophotometer (Shimadzu). Absorbance was measured at wavelength (λ) of 540 nm [17]. Standard curves were generated and depicted in Fig. 1. Adsorption efficiencies were calculated using following formula:

$$ H=\frac{C_{\mathrm{o}}-{C}_{\mathrm{e}}}{C_{\mathrm{o}}}\times 100\ \left(\%\right) $$

where

*H*: Adsorption efficiency (%)

*C*_o_: Initial concentration (mg/L)

*C*_e_: Equilibirium concentration (mg/L)

### Method for determining the isotherm adsorption equations

*Freundlich adsorption model*: The Freundlich model is used to describe the adsorption model from liquids and can be expressed as the following equation [17]:

$$ \ln {q}_{\mathrm{e}}=\ln {K}_{\mathrm{F}}+\frac{1}{n}\times \ln {C}_{\mathrm{e}} $$

*Langmuir adsorption model*: The Langmuir model, which is mainly used to determine the maximum adsorption capacity, is expressed as the following equation [17]:

$$ \frac{C_e}{q_{\mathrm{e}}}=\frac{1}{q_{\mathrm{max}}}\times {C}_{\mathrm{e}}+\frac{1}{q_{\mathrm{max}}\times {K}_{\mathrm{L}}} $$

where:

*C*_e_: concentration at equilibrium stage (mg)

*q*_e_: adsorption capacity at equilibrium stage (mg/g)

*q*_max_: maximum adsorption capacity (mg/g)

*K*_L_: adsorption constant for Langmuir (L/mg)

*K*_F_, 1/n: adsorption constants for Freundlich (L/mg)

### Statistical analysis

In this study, all experiments were repeated three times, and the collected data were analyzed with the appropriate statistical tests. To compare the two groups, the Mann-Whitney *U* test (for non-parametric comparisons) or Student’s *t* test (for parametric comparisons) were used. Significance was set at three levels with *P* < 0.05 [1, 2].

## Results

### Synthesis of spherical melanin beads

**Fig. 2:**
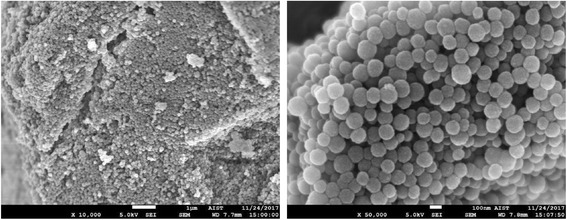
Morphology and purification of melanin isolated from squid ink sacs were examined under scanning electron microscope (SEM) at × 10,000 (left image) and × 50,000 (right image)

After purification, isolated melanin pellet was lyophilized to obtain the intact natural squid melanin. Then the melanin sample was examined by the scanning electron microscope (SEM), which showed high purity without contamination by any cellular components (Fig. 2). This purified melanin was more than enough for treatment of heavy metal ions in adsorption experiments [18].

**Fig. 3:**
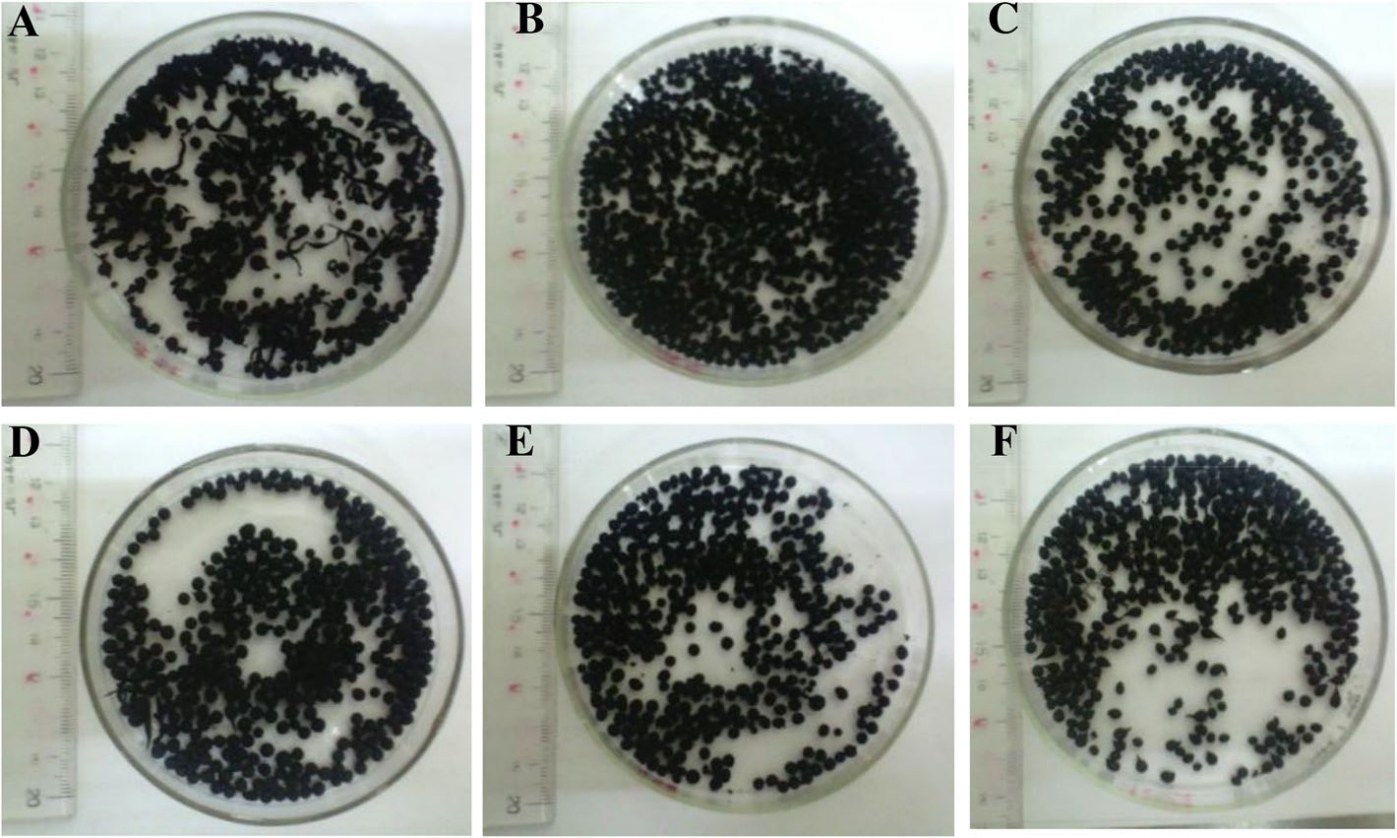
Melanin-embedded beads with **a** 3% alginate, **b** 4% alginate, **c** 5% alginate, **d** 7% alginate, **e** 10% alginate, and **f** 15% alginate as binding agent

To produce the spherical melanin-embedded beads, melanin powder was added into the binding agent solution containing alginate at different percentages, which varied from 3 to 15%, to form a mixture before dropping into the CaCl_2_ solution to form beads (Fig. 3). The results showed that at low percentages of alginate (3 and 4%), the formed melanin beads were not stable and were easily broken since the concentration of the binding agent was insufficient. At the high percentages of alginate (12 and 15%), the formed melanin beads did not have spherical shape because the viscosity of the mixture was too high. Percentages of alginate in the range of 5–10% were optimal to form stable melanin beads with spherical shape (Fig. 3).

**Fig. 4:**
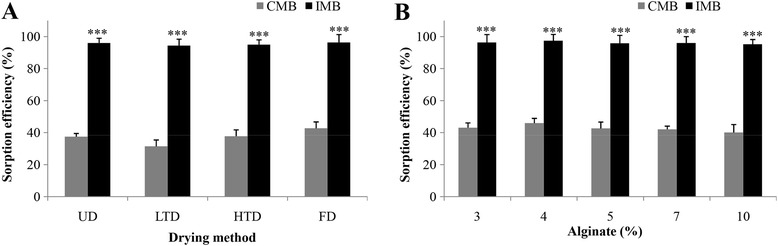
Effect of drying method (**a**) and alginate content (**b**) on sorption efficiency of Cr^VI^ on IMB and CMB. (UD: undrying; LTD: low temperature drying; HTD: high temperature drying; FD: freezing drying). Three asterisks indicate significant difference (*p* < 0.001) between IMB and CMB by the Student’s *t* test

In addition, neither alginate content (in the range of 3–15%) nor the drying method (un-drying, low-temperature drying, high-temperature drying, or freezing drying) had any significant effect on the sorption capacities of the beads (Fig. 4). However, alginate at 5% was chosen because it yielded the highest productivity and uniformity of the melanin beads.

### Effect of pH on Cr^VI^ sorption by melanin-embedded beads

**Fig. 5:**
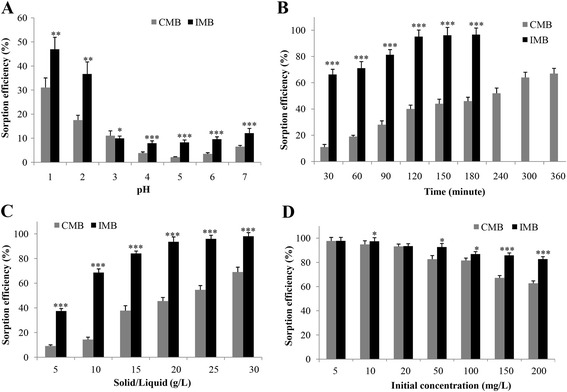
Effect of pH (**a**), sorption time (**b**), solid/liquid (**c**), and Cr^VI^ initial concentration (**d**) on sorption efficiency of Cr^VI^ on CMB and IMB. *, **, and *** Significant difference (*p* < 0.05, 0.01 and 0.001, respectively) between IMB and CMB by the Student’s *t* test

The effect of pH on the efficiency of Cr^VI^ removal by melanin beads was evaluated for the following set conditions: shaking rate of 200 rpm at 30 °C, solid/liquid ratio of 10 g/L, shaking time of 1 h, and Cr^VI^ initial concentration of 200 mg/L. The results are shown in Fig. 5. The removal efficiencies of Cr^VI^ by IMB or CMB were better at lower pH values and reached the maximum at pH 1–2 (Fig. 5a). However, IMB had a much higher sorption capacity compared to that of CMB at any pH value. In particular, at the optimized pH (1–2), the sorption capacity of IMB was almost threefold higher than that of CMB.

### Effect of sorption time on Cr^VI^ removal efficiency

The effect of sorption time on the efficiency of Cr^VI^ removal by melanin beads was also evaluated for the following set conditions: pH of 2, shaking rate of 200 rpm at 30 °C, initial Cr^VI^ concentration of 200 mg/L, and the solid/liquid ratios of 20 g/L. The results indicated that the longer the sorption time, the higher the removal efficiency. However, the removal efficiency quickly increased during the first hour then slowly increased and reached the highest values around 96% at 2 h for IMB and 67% at 6 h for CMB (Fig. 5b). In general, at any sorption time, IMB was more effective than CMB at removing Cr^VI^. In particular, at the same sorption time of 2 h, the sorption capacity of IMB was 2.8-fold higher than that of CMB.

### Effect of solid/liquid ratios on sorption efficiency

To investigate the effect of solid/liquid ratios on the efficiency of Cr^VI^ adsorption, we tested solid/liquid ratios in the range of 1–30 g/L with the following set conditions: pH of 2, shaking rate of 200 rpm at 30 °C, shaking time of 1 h, and Cr^VI^ initial concentration of 200 mg/L. The results showed that the removal efficiency increased rapidly as the solid/liquid ratio increased from 1 to 20 g/L and increased only slightly from 20 to 30 g/L. The maximum removal efficiencies reached 95 and 35% for IMB and CMB, respectively, at the solid/liquid ratio of 30 g/L (Fig. 5c). At the same solid/liquid ratio, IMB was much more effective than CMB at eliminating Cr^VI^.

### Effect of initial concentration of Cr^VI^ on sorption efficiency

The effect of the initial concentration of Cr^VI^ on the efficiency of Cr^VI^ removal by melanin beads was evaluated for the following set conditions: pH of 2, shaking rate of 200 rpm at 30 °C, solid/liquid ratio of 20 g/L, and sorption time of 2 h for IMB or 4 h for CMB. The initial concentrations of Cr^VI^ were in the range of 5–200 mg/L. The Cr^VI^ removal efficiencies and the sorption capacities of CMB and IMB are shown in Fig. 5d and presented in Tables 1 and 2. The maximum capacities for IMB and CMB were 19.6 and 6.24, respectively. These results indicate that while CMB is not that efficient at eliminating Cr^VI^, IMB is efficient and serves as a promising material for Cr^VI^ removal due to its high sorption capacity, especially as bead form. Previous studies have tested numerous materials (e.g., activated carbon, sludge, plant-leaf materials, and chitosan gel) for Cr^VI^ removal from aqueous solution and have shown that these materials as powder form had sorption capacities of wide range from 6 mg/g to 50 mg/g [3, 4]. Our study shows that IMB (in bead form) is a highly effective material for removing Cr^VI^ in water.

**Table 1:** Cr^VI^ removal efficiencies and sorption capacities of CMB

Initial conc. (mg/L)	Output conc. (mg/L)	Cr^VI^ removal efficiency (%)	Sorption capacity (q) (mg/g)
5	0.15	97.7	0.32
10	0.57	94.9	0.53
20	1.46	93.2	1.00
50	9.48	82.7	2.26
100	19.1	81.6	4.22
150	46.0	67.2	4.73
200	74.3	62.8	6.24

**Table 2:** Cr^VI^ removal efficiencies and sorption capacities of IMB

Initial conc. (mg/L)	Output conc. (mg/L)	Removal efficiency (%)	Sorption capacity (q) (mg/g)
5	0.13	97.8	0.50
10	0.26	97.5	0.99
20	1.27	93.5	1.83
50	3.5	92.7	4.44
100	12.5	86.9	8.31
150	20.2	85.9	12.2
200	24.7	82.8	19.6

### Cr^VI^ sorption kinetics

**Fig. 6:**
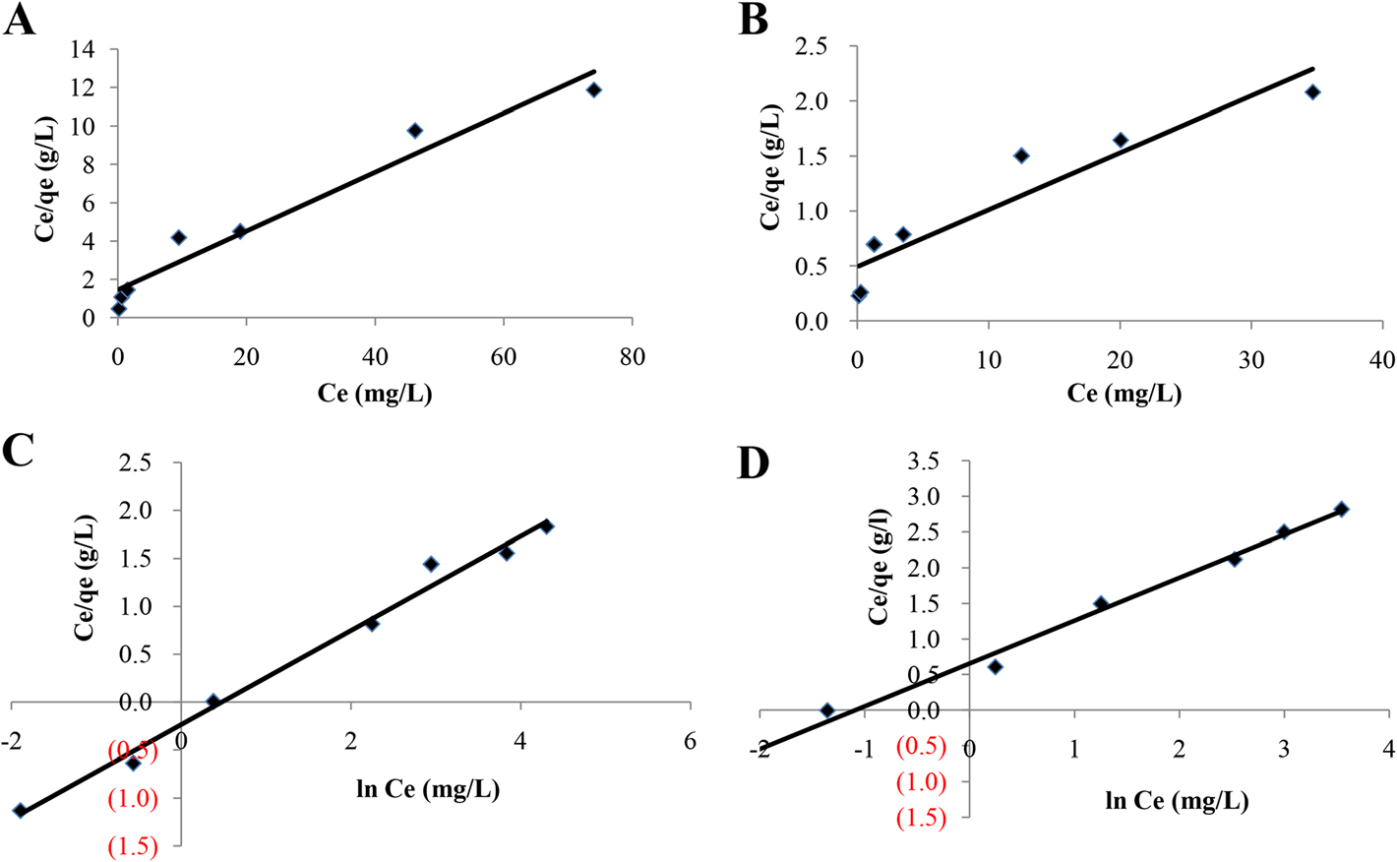
Langmuir model (**a** and **b**) and Freundlich model (**c** and **d**) for isotherm sorption mechanisms of Cr^VI^ on CMB (**a** and **c**) and IMB (**b** and **d**)

The results of this study indicate that an increase of initial concentration can lead to a decrease of Cr^VI^ removal efficiency and increase of the sorption capacity. From the isotherm adsorption results of Cr^VI^ at different concentrations on IMB and CMB at optimized conditions, we then examined the suitable isotherm adsorption model for adsorption of Cr^VI^ on IMB and CMB using the two common models of Langmuir and Freundlich. The results are shown in Fig. 6. The isotherm equations deduced from Langmuir and Freundlich models were presented as follows:

Equation of the Langmuir model for CMB (Fig. 6a): $\frac{C_e}{q_e}$ = 0.153 C_e_ + 1.450; *R*^2^ = 0.955

Equation of the Langmuir model for IMB (Fig. 6b): $\frac{C_{\mathrm{e}}}{q_{\mathrm{e}}}$ = 0.051 C_e_ + 0.492; *R*^2^ = 0.885

Equation of the Freundlich model for CMB (Fig. 6c): ln *q*_e_ = 0.491lnC_e_ − 0.237; *R*^2^ = 0.989.

Equation of the Freundlich model for IMB (Fig. 6d): ln *q*_e_ = 0.601lnC_e_ + 0.655; *R*^2^ = 0.992

Parameters for isotherm adsorption of CMB and IMB are summarized in Table 3. The results suggest that the Freundlich model is more suitable than the Langmuir model to describe the sorption mechanism of Cr^VI^ on melanin bead since the *R*^2^—coefficient value of the Freundlich model—was higher than that of the Langmuir model. The data also indicate that the surfaces of IMB or CMB are not uniform, and therefore, the distributions of reaction centers on the surface of the materials probably follow an exponential equation rather than a linear one. In the Freundlich model, the mechanism and the rate of adsorption are functions of the constants 1/n and K_F_. For a good absorbance, the 1/n value should be 0.2 < 1/*n* < 0.8, and a smaller value of 1/n indicates better adsorption and formation of strong bonds between the adsorbate and adsorbent [19, 20]. In this study, the 1/n values of 0.49 and 0.6 for CMB and IMB, respectively, demonstrate that both IMB and CMB are good materials for adsorption of Cr^VI^; however, IMB has a much better adsorption capacity compared to CMB.

**Table 3:** Parameters for isotherm sorption of CMB and IMB materials

	Langmuir constants			Freundlich constants		
	*q*_max_	*K*_L_	*R* ^2^	K_F_	1/n	*R* ^2^
CMB	6.536	0.106	0.955	0.789	0.49	0.989
IMB	19.608	0.104	0.885	1.925	0.60	0.992

### Fourier transform infrared analysis

**Fig. 7:**
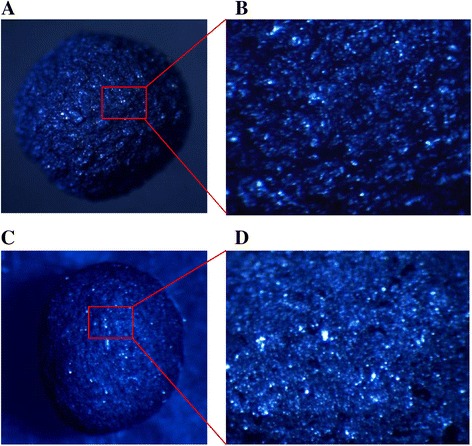
Surface structures of IMB (**a** and **b**) and CMB (**c** and **d**) at × 10 magnifications

In general, on the surface of the melanin material, there are many chemical groups including hydroxyl, carboxyl, and ether, which have been proposed to be responsible for sorption of metal ions by formation of chemical bonding. The chemical-sorption ability of the material depends on factors such as quantity of active centers, its accessibility, and affinity between active centers and metal ions [21]. The surfaces of IMB and CMB were observed under stereo-microscope and presented in Fig. 7. The differences in surface structure of IMB and CMB are clearly distinguishable. It showed that the intensities of peaks of the hydroxyl, carboxyl, and ether groups in IMB were very clear and sharp, while the intensities of these corresponding peaks in CMB were not so clear especially for hydroxyl group. This result indicated that the distribution of chemical groups on the surface of IMB may be denser on the surface of CMB and might lead to difference in numbers of chemical linkages formed between melanin and Cr^VI^ ion.

**Fig. 8:**
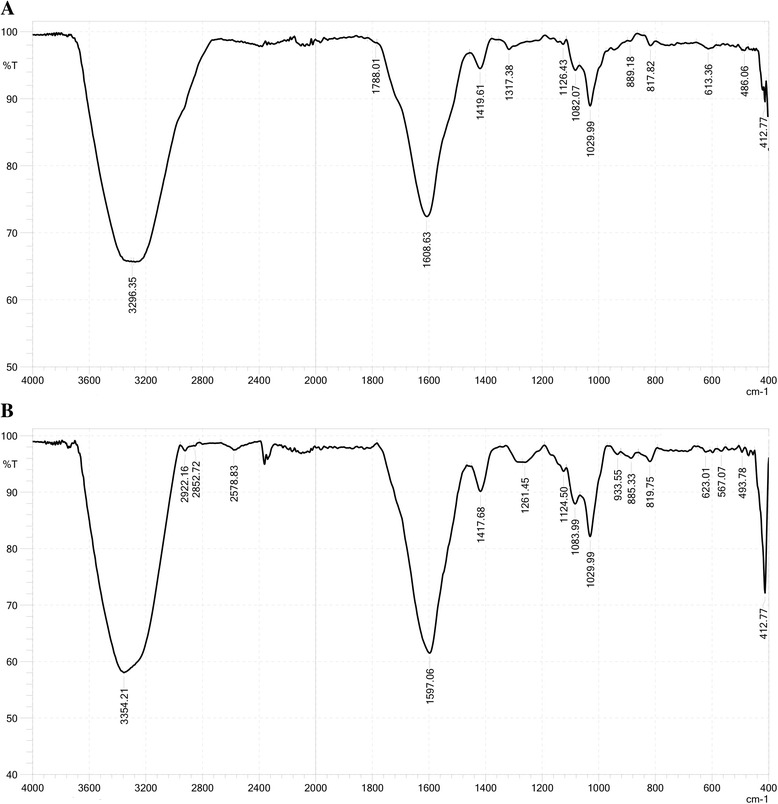
FTIR spectra of native IMB (**a**) and Cr^VI^-loaded IMB (**b**)

**Fig. 9:**
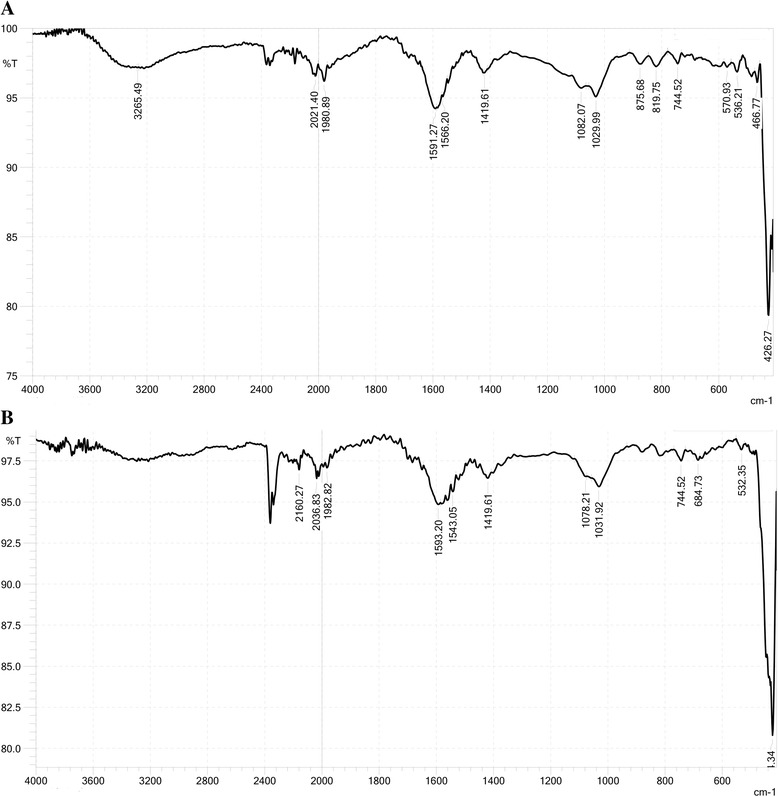
FTIR spectra of native CMB (**a**) and Cr^VI^-loaded CMB (**b**)

Conversely, FTIR analysis was used to analyze the functional groups on the surfaces of the native and Cr^VI^-bound IMB and CMB; results are shown in Figs. 8 and 9. IMB and CMB showed completely different FTIR spectra. While IMB had the broad absorption peaks at 3296 cm^− 1^ and 1608 cm^− 1^ due to the presence of the –OH and –C=O groups, respectively [22, 23], there were almost no peaks at these sites on the surface of CMB (Fig. 8a and Fig. 9a). Although many other sorption peaks were observed, it is difficult to interpret all. After loading Cr^VI^, the FTIR spectra of Cr^VI^-bound IMB and Cr^VI^-bound CMB were presented in Fig. 8b and Fig. 9b. The results indicated that the adsorption of Cr^VI^ on the surface of IMB may have caused a shift of the broad peaks at 3296 cm^− 1^ and 1608 cm^− 1^ to 3354 cm^− 1^ and 1597 cm^− 1^, respectively, due to –OH and –C=O stretching (Fig. 8b).

## Discussion

Chromium pollution originated from plating and electroplating industries, iron and steel industries, and inorganic-chemical production represents a huge problem for environmental health [24]. Exposure to chromium ions, especially Cr^VI^, may cause diseases related to the digestive system and lung; such complications can include epigastric pain, nausea, diarrhea, hemorrhage, and cancer [25]. Thus, it is essential to eliminate Cr^VI^ from wastewater before disposal. There are many methods which can be applied to remove Cr^VI^ from aqueous solutions; these methods include ion exchange [26], chemical precipitation [27], electrochemical precipitation [28], reduction [29], solvent extraction [30], adsorption [31], membrane separation [32], and reverse osmosis and biosorption [33]. However, these different methods have different disadvantages, such as low removal efficiency, expensive equipment, high operating cost, and high energy requirement [34].

In this study, we investigated the ability of melanin (as a material in bead form) to remove Cr^VI^ from aqueous solution. Two different natural melanin sources, one originating from plant (commercial one) and the other extracted from ink sacs of squid (isolated one), were used for making melanin-embedded beads; the beads were called CMB and IMB, respectively. In many Asian countries, the seafood industry is one of the most important industries which provide great economic benefit for the country. Squid and octopuses are processed in many seafood processing companies for export. Nevertheless, ink sacs of squid and octopuses are wastes in these seafood companies. More importantly, melanin accounts for about 16–18% in total weight of the sac [14]. Thus, utilization of these wastes for melanin production will have great impact since melanin has not only been considered as a potential material for heavy metal removal but also for many other applications, such as medicine and cosmetics [6–9].

To examine the effect of IMB and CMB on removing Cr^VI^, the effect of various parameters such as pH, sorption time, and solid-liquid ratio on Cr^VI^ sorption were conducted, and isotherm models including Freundlich and Langmuir were applied to fit experimental data. In accordance with previous studies [19, 35–39], the data showed that IMB and CMB both had the highest sorption capacities as pH 1–2 and that the Freundlich model was the best model to represent the sorption model of Cr^VI^ on IMB and/or CMB.

There are many materials which have been used to remove chromium ions in effluents from various industries. The removal capacities of these materials vary from 0.2 to 200 mg/g. In general, sorption capacities of materials are different from their origins, for example: plant-originated materials (0.5–10 mg/g), activated carbon materials (2–30 mg/g), coal (6.68 mg/g), hydrous titanium oxide (5 mg/g), maghemite nanoparticles (1.5 mg/g), and tannin gel (200 mg/g) [19, 35–39]. In addition, almost all materials used in previous studies were in powder form, and therefore, their capacities in Cr^VI^ removal would be significantly decreased after making the bead form. Previous studies demonstrated that acidic conditions at pH of 1 or 2 were good for the removal of Cr^VI^ from water [35–39]. This study also introduced the similar result. In practical conditions, Cr^VI^ pollutant mostly comes from the mining and platting industries, which normally have effluents with low pH values. That means IMB should be a suitable material for treatment of Cr^VI^ from industrial effluent. Besides that, in some cases, Cr^VI^ pollutant may also come from natural water, which has pH of 5–7. However, concentration of Cr^VI^ in natural water is below 1 μg/L [40], while this study showed that the adsorption capacity of IMB for Cr^VI^ was about 7–8 mg/g at the pH of 6–7 (one third of that at pH of 1–2). It means that IMB is also good enough for removing of Cr^VI^ from natural water.

## Conclusions

In this study, our results demonstrated that melanin materials are potential for the removal of Cr^VI^ from water. However, melanin from different sources have different physical and chemical properties. Particularly, the properties of IMB (melanin extracted from squid ink sacs) were significantly different from those of CMB (melanin extracted from plant). These results led to a difference in the ability of these two melanin materials to eliminate Cr^VI^ from aquaous solution. CMB had Cr^VI^ sorption capacity of 6.24 mg/g while IMB had Cr^VI^ sorption capacity of 19.8 mg/g. In summary, our study suggests that melanin isolated from squid ink sacs (which are considered as waste of seafood processing companies) can be used to synthesis the melanin bead and applied in water treatment to effectively remove Cr^VI^ ions.

## Abbreviations

CMB: Commercial Melanin Bead
Cr^VI^: Hexavalent chromium
FTIR: Fourier-transform infrared analysis
IMB: Isolated Melanin Bead
